# Growth Hormone Protects the Intestine Preserving Radiotherapy Efficacy on Tumors: A Short-Term Study

**DOI:** 10.1371/journal.pone.0144537

**Published:** 2015-12-15

**Authors:** Victor Caz, Marcos Elvira, Maria Tabernero, Antonio G. Grande, Bricia Lopez-Plaza, Enrique de Miguel, Carlota Largo, Monica Santamaria

**Affiliations:** 1 Experimental Surgery Department, La Paz Hospital Research Institute, Madrid, Spain; 2 Department of Radiation Oncology, La Paz University Hospital, Madrid, Spain; 3 Department of Nutrition, La Paz Hospital Research Institute, Madrid, Spain; Center for Cancer Research, National Cancer Institute, UNITED STATES

## Abstract

The efficacy of radiotherapy on tumors is hampered by its devastating adverse effects on healthy tissue, particularly that of the gastrointestinal tract. These effects cause acute symptoms that are so disruptive to patients that they can lead to interruption of the radiotherapy program. These adverse effects could limit the intensity of radiation received by the patient, resulting in a sublethal dose to the tumor, thus increasing the risk of tumor resistance. The lack of an effective treatment to protect the bowel during radiation therapy to allow higher radiation doses that are lethal to the tumor has become a barrier to implementing effective therapy. In this study, we present a comparative analysis of both intestinal and tumor tissue in regard to the efficacy and the preventive impact of a short-term growth hormone (GH) treatment in tumor-bearing rats as a protective agent during radiotherapy. Our data show that the exogenous administration of GH improved intestinal recovery after radiation treatment while preserving the therapeutic effect against the tumor. GH significantly increased proliferation in the irradiated intestine but not in the irradiated tumors, as assessed by Positron Emission Tomography and the proliferative markers Ki67, cyclin D3, and Proliferating Cell Nuclear Antigen. This proliferative effect was consistent with a significant increase in irradiated intestinal villi and crypt length. Furthermore, GH significantly decreased caspase-3 activity in the intestine, whereas GH did not produce this effect in the irradiated tumors. In conclusion, short-term GH treatment protects the bowel, inducing proliferation while reducing apoptosis in healthy intestinal tissue and preserving radiotherapy efficacy on tumors.

## Introduction

Radiotherapy is currently used for over 70% of cancer patients during the course of their treatment [1]. Its usefulness is limited, however, by the high incidence of radiation-induced adverse effects, which primarily affect tissues with high cellular turnover, such as tissues of the gastrointestinal tract, bone marrow, and skin [2]. Specifically, abdominal irradiation can produce radiation enteritis, and recent reports indicate that as many as 75% of those receiving radiotherapy suffer significant adverse effects from this therapy [3,4]. As a result, the radiation dose is reduced—or in extreme cases interrupted—to preserve the patient’s status, thus decreasing radiotherapy efficacy and inducing tumor resistance. To prevent this loss of efficacy, much effort has been made in the past 5–10 years to develop medical countermeasures against the adverse effects of radiation, and many of these efforts have been directed toward reducing gastrointestinal radiation damage [1,2,5].

Several approaches have been investigated to reduce the negative impact of anticancer therapy on the intestine, including dietary modification [6] and the use of trophic factors [3,7]. We have previously shown the beneficial effects of GH treatment for acute radiation-induced injury in the small intestine of rats exposed to a sublethal dose of radiation. These beneficial effects were attributed to the proliferative and antiapoptotic effects of GH on the ileal crypts [8]. In the irradiated ileum of rats, we have shown that GH administration upregulates mRNA and protein expression of intestinal trefoil factor [9], a protective peptide against radiation-induced intestinal mucositis [10]. Other authors have shown additional positive GH effects: Specifically, GH counteracts the loss of progenitor cells in irradiated bone marrow [11] and GH administration selectively augments the early-outgrowth endothelial progenitor cell population in healthy individuals, indicating possible implications for the use of GH in future regenerative cell-based therapies [12]. Furthermore, the use of GH delays and decreases the severity of radiation-induced dermatitis [13]. The benefits of GH as an anticachectic agent have also been reported [14], stimulating liver protein synthesis in an animal model without changing tumor growth [15].

Despite the benefits related to these GH therapies, their effects on irradiated tumors are still poorly understood. The studies on these therapies’ effects on nonirradiated tumors are controversial, primarily because of their potential effect on the proliferation and survival of cancer cells [16,17].

The objective of this study was to investigate whether GH treatment would protect the intestine against acute injury induced by radiation without impairing the therapeutic effects of radiotherapy in terms of cell viability, cell proliferation, and apoptosis.

## Methods and Materials

### Cell culture and *in vitro* analysis

The rat cell lines, DHD/K12/Trb (colonic adenocarcinoma, 90062901; DHD) and MCA-RH 7777 (hepatocarcinoma, 90021504; RH), were obtained from the European Collection of Cell Cultures (ECACC) and were cultured according to their instructions.

To assess cell proliferation, cells (50–150 x10^3^) were seeded into 6-well culture plates in standard culture medium and treated or not with GH (0.2 μg/ml). At the indicated times (at least 3 wells per time point), the cells were harvested with trypsin, diluted in medium with 10% fetal bovine serum, and centrifuged. The cell pellets were resuspended in 100 μL of medium and counted twice in a Neubauer chamber.

### *In vivo* experiments

This study was performed in strict accordance with the recommendations from the European Union Criteria for Animal Use in Scientific Experimentation (63/2010 EU) and related Spanish legislation (RD 53/2013). The protocol was approved by the Animal Welfare Ethics Committee of La Paz University Hospital (Permit number: 04–2006). All surgery was performed under isoflurane anesthesia, and all efforts were made to minimize suffering.

Twenty-five adult BDIX rats (Charles River Laboratories, France) and 25 adult Buffalo rats weighing 225–250 g (Harlan Laboratories, Netherlands) were used. The animals were housed in normal conditions and received standard food and water *ad libitum*. All the *in vivo* experiments were performed on both rat strains.

**Fig 1:**
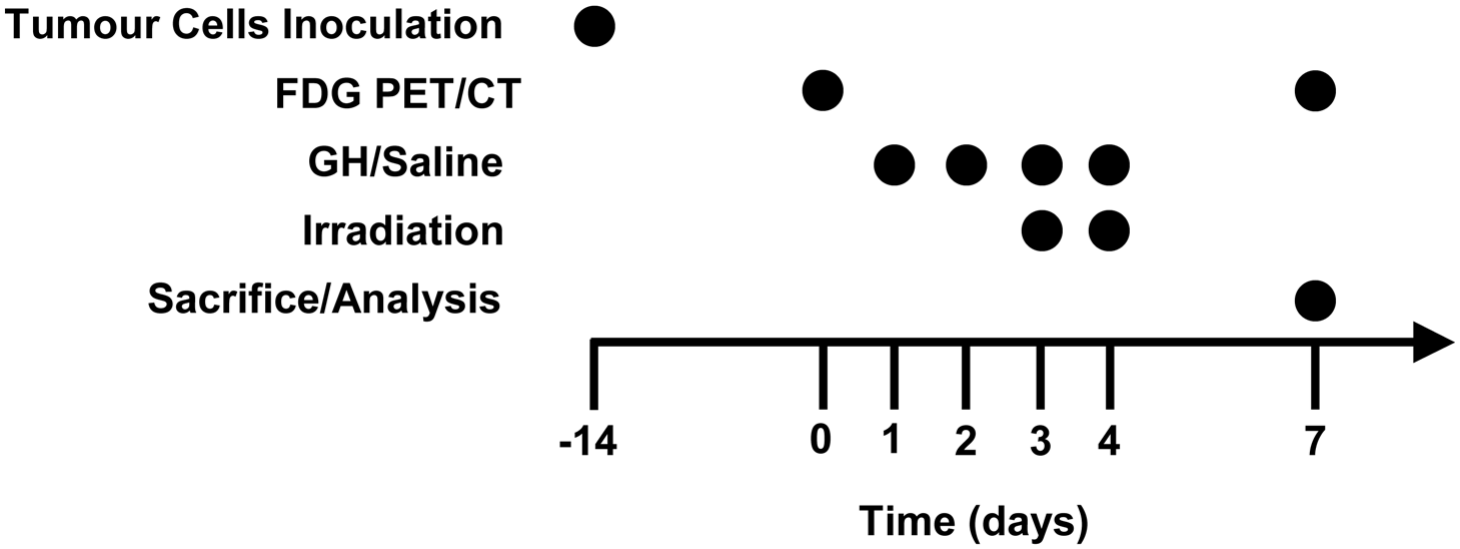
Scheme of the study’s experimental design. Tumor cells (DHD or RH cells) were inoculated subcutaneously (1x10^6^ tumor cells/rat) and allowed to develop for 14 days (day 0). At this time, the rats were subjected to the first FDG PET/CT, to have a baseline of the animals’ metabolic status, both in the intestine and the tumors. At day 1, the rats bearing tumors were divided into 3 groups of 7 animals, for the respective treatments on days 1 to 4: the control and irradiated group received saline; and the third group received recombinant human GH (1 mg/kg/day). On days 3 and 4, all the animals except the control group were exposed to 6 Gy abdominal irradiation (0.92 Gy/min). At 72 h after the last irradiation (day 7), the animals were subjected to a second FDG PET/CT and euthanized.

The experimental design and chronology of the *in vivo* experiments are shown in Fig 1. Briefly, the DHD or RH tumor cells were inoculated subcutaneously in the middle to lower abdomen of the BDIX and Buffalo rats, respectively (1x10^6^ tumor cells/rat) and allowed to develop for 14 days. At this time (day 0), the animals were subjected to Positron Emission Tomography (PET) to obtain baseline imaging (see the molecular imaging studies section).

The rats bearing tumors were divided into 3 groups of 7 rats as follows: the control group (C) received a subcutaneous injection of 50 μL saline on days 1 to 4; the IR group (IR) received saline as above and fractionated Ɣ-irradiation; the GH+IR group (GH_IR) received Ɣ-irradiation as above and recombinant human GH (Pfizer, Spain) 1 mg/kg/day subcutaneously injected on days 1 to 4. On days 3 and 4, GH was administered one hour before radiotherapy.

The animals were exposed to two doses of 6 Gy abdominal irradiation (0.92 Gy/min, ^60^Co) on day 3 and 4 under anesthesia with 2% isoflurane, avoiding liver exposure and using a telecobaltotherapy unit (model Theratron-80, Theratronics). Three days after radiotherapy, the animals were subjected to a second PET. The animals were then laparotomized and euthanized with an intracardiac overdose of 1M KCl. Ileum samples were collected 1 cm from the ileocecal valve. One part was processed for immunohistochemistry and the other part was frozen at -80°C for determination of biochemical parameters. The tumor samples (colonic adenocarcinoma: ACC; hepatocarcinoma: HCC) were processed in the same way.

### Immunohistochemistry

Growth hormone receptor (GHR) was detected by immunohistochemistry using an anti-GHR monoclonal antibody (Santa Cruz Biotechnology, CA, USA) in the ileum, cells, and tumor sections as previously described [18], and was measured using a semi-quantitative scoring system. Human IgG was used instead of primary antibody as a negative control. The staining intensity score of the cells was graded as negative (0), faint yellow staining (1), brown staining (2), or dark brown staining (3). The percentage of positive cells was graded as 0% (0), 0%–10% (1), 10%–50% (2), and 50%–100% (3). The GHR expression score was calculated by multiplying the two scores, and is indicated as arbitrary units. Ten microscopic fields (200x) were observed for each section.

Ki67 expression was assessed in the ileum and tumor sections with anti-Ki67 antibodies (Novo Castra Laboratories, UK) and then with a biotinylated goat anti-mouse antibody (Santa Cruz Biotechnology, CA, USA). Ten microscopic fields (200x) were observed for each section. The results are expressed as the percentage of Ki67 positive cells.

### RNA isolation and analysis of gene expression

Total RNA was extracted using TRI Reagent (Sigma). The reverse transcription and real-time polymerase chain reaction (PCR) was performed using an iCycler (Bio-Rad, Hercules, CA) and the iQ SYBR Green Supermix (Bio-Rad) as previously described [19]. The following primers were used: sense 5’-ATGACTCTACCCACGGCAAG-3’ and antisense 5’-GGAAGATGGTGATGGGTTTC-3’ for GAPDH; sense 5’-GAAATAGTGCAACCTGATCCGCCCA-3’ and antisense 5’-GCGGTGGCTGCCAACTCACT-3’ for GHR; sense 5’-CACAGCATCTCCAATATGGC-3’ and antisense 5’-ACTTGGAATCCCAGAACAGG-3’ for proliferating cell nuclear antigen (PCNA); sense 5’-CTTCCCTCTGGCTATGAACTACCT-3’ and antisense 5’-AAGCTGCAGTTGCGCCTTT-3’ for cyclin D3; 5’-ACAGCGTGGTGGTACCGTAT-3’ and antisense 5’-GGAGCTGTTGCACATGTACT-3’ for p53; and 5’-CCGGGAGAACAGGGTATGATAA-3’ and antisense 5’-CCCACTCGTAGCCCCTCTG-3’ for Bcl-2.

### Morphometric analysis of the small intestine

The lengths of the villi and the intestinal crypts were measured in hematoxylin-eosin stained sections, and 10 fields were analyzed in each animal. The statistical analysis considered the mean of these measurements—at least 10 villi and 25 crypts per animal—as a single datum.

### Analysis of caspase-3 activity in intestinal and tumor tissues

Caspase-3 activity was measured in the ileal mucosa (stripped of muscular and serosal layers) and the tumor tissue samples using a caspase-3-specific fluorogenic substrate (Ac-DEVD-AMC, BD Biosciences, Belgium) as previously described [20].

### Apoptosis analysis by flow cytometry

To analyze radiation-induced apoptosis in the tumor cells, DHD and RH cells were seeded (10^4^/cm^2^), treated with GH on days 1 to 4 (0.2 μg/mL), and irradiated on days 3 and 4 with 6 Gy/day (X-rays, 1.05 Gy/min). The groups were the same as the animal models. The cells were harvested 4, 8, 16, and 24 h after radiation, and processed as previously described [21]. Briefly: fixed in methanol-phosphate buffered saline (PBS) (9:1), washed with PBS, incubated with 25 μg/ml RNAse A (Sigma) for 15 min at room temperature, and stained with 50 μg/ml propidium iodide (Sigma) for 30 min at 4°C. The samples were analyzed on a FACSCalibur flow cytometer (Becton Dickinson, CA, USA). A minimum of 1 x 10^4^ cells was analyzed for each sample. Cell cycle phase distributions were determined using CellQuestPro software (Becton Dickinson) and are expressed as percentages.

### Clonogenic survival assay

Exponentially growing cells were maintained in a basal unstimulated state (C) or pretreated with GH (0.2 μg/mL) for 24 h. After treatment, both the DHD and RH cells were harvested with trypsin and seeded in triplicates onto 6-well-plates following appropriate dilutions according to the size and proliferation rate (200, 1000, and 5000 cells/well for RH; 100 and 1000 cells/well for DHD) and allowed to attach overnight. Four experimental groups were defined: unstimulated control cells (C), irradiated nontreated cells (IR), cells treated with GH before irradiation (GH_IR), and cells irradiated and treated with GH following RT (IR_GH). Radiation treatment was performed on the following day with 0, 2, 4, or 6 Gy, and GH was maintained for the GH_IR group until the end of the RT (36 hours of GH treatment). Immediately after RT, the cells were washed, the medium replaced, and the IR_GH group treated with GH for 36 h. Twelve days after seeding, the colonies were rinsed with PBS, fixed with glutaraldehyde (Sigma-Aldrich, St. Louis, MO, USA), and stained with 1% crystal violet dye (Sigma-Aldrich). The number of colonies containing at least 50 cells was digitally determined using ImageJ software (National Institutes of Health, Bethesda, MD) with customized macros. Plating efficiency (PE = number of colonies formed/number of cells seeded) and survival factor (SF = number of colonies after treatment/number of cells seeded x PE) were determined for each cell line and treatment. The curves were normalized to control for unstimulated nonradiated cells (100% cell survival).

### Molecular imaging studies: [^18^F]-FDG PET/CT

PET imaging using 2-Deoxy-2-[^18^F] fluoro-D-glucose (FDG) with computed tomography (CT) attenuation correction was performed in the eXplore VISTA dual-ring scanner (GE Healthcare). FDG (37 MBq) was administered into the tail vein and the rats were anesthetized 45 minutes later with 1.5% isofluorane, when FDG uptake was complete and a steady state was reached. The small intestine and the tumor response to radiotherapy were evaluated *in vivo* using two FDG PET/CT studies: the first was performed on day 0 under baseline conditions and the second was performed on day 7, three days after radiotherapy. FDG uptake was semi-quantitatively determined by two independent experts in a blind study using the mean Standardized Uptake Value (SUV). The small intestine and tumor SUV values were normalized with non-irradiated liver SUV values, because normal liver has low inter-patient SUV variability, as described elsewhere [22]. Given the possibility that the lower part of the liver could be exposed to abdominal irradiation, the upper part of the liver was selected to prevent this limitation.

### Statistical analysis

The data are presented as mean values ± standard deviation (SD). A 2-tailed unpaired t-test was used to compare 2 groups of values when n was greater than 10. For smaller groups, the Mann-Whitney nonparametric test was used. The analyses were performed using GraphPad Prism version 5.00 (GraphPad Software, San Diego, CA). Significance was set at *P* < .05.

For the clonogenic survival assay, the Chi-squared test was used to examine the relationship between quantitative variables. The Kolmogorov-Smirnov test was used to determine the normal distribution of data. Mean and standard deviations (SD) were calculated for the continuous variables. A general linear model (MANOVA multivariate analysis) was used to determine the survival factor from DHD and RH in relation to the treatment groups and the radiation dose. Multiple ANOVA comparisons were made employing the Bonferroni post hoc correction. All the analyses were performed using SPSS v.17.0 software (SPSS Inc., 272 Chicago, IL, USA). Significance was set at *P* = .05.

## Results

### GH treatment modulates GHR expression but not proliferation in cultured cells

**Fig 2:**
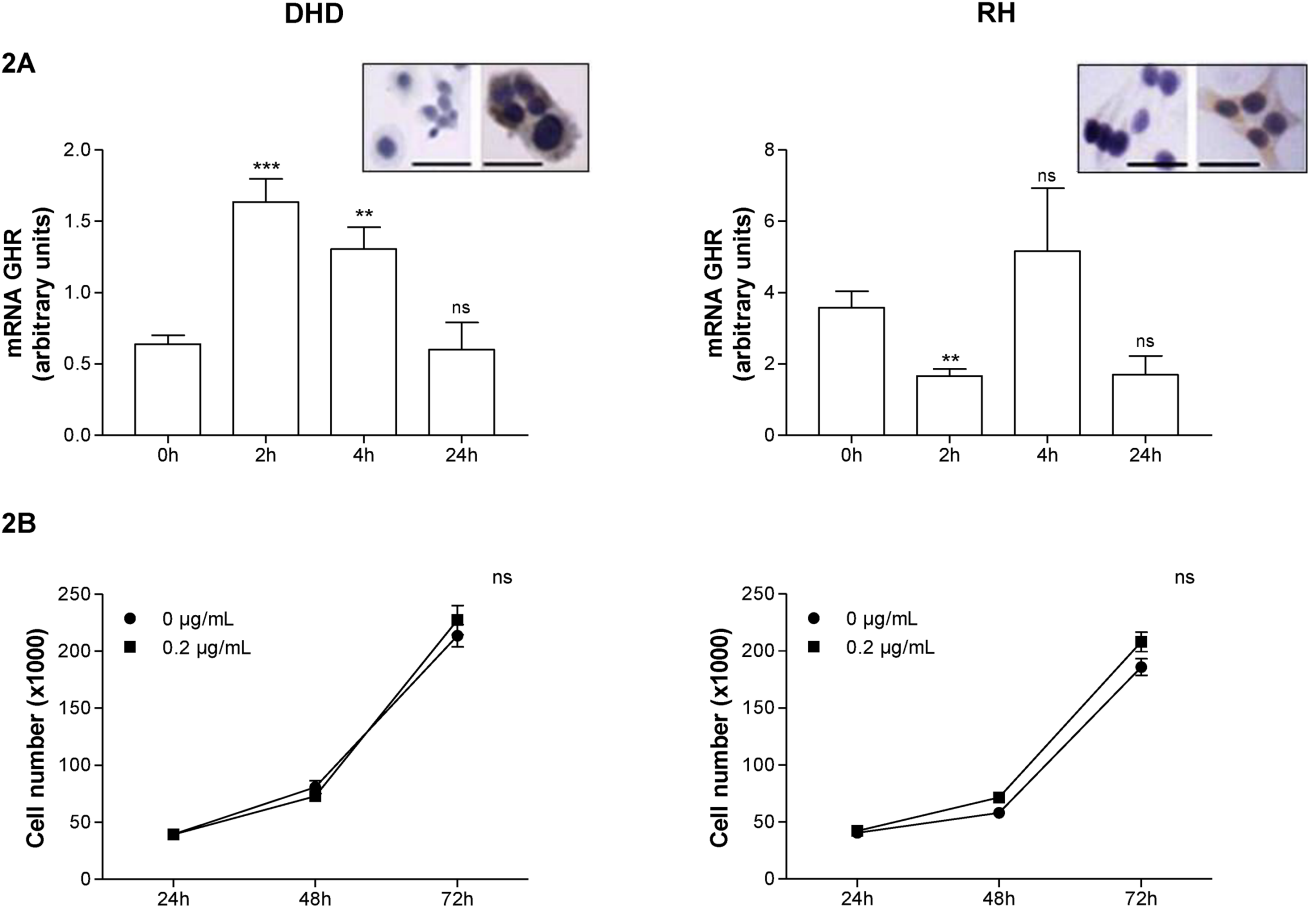
Effect of GH treatment on GHR expression and proliferation of DHD and RH cells. (A) Expression of the receptor (GHR) in DHD and RH cell lines. Data are mean ± standard deviation of the cell line cultures. GHR mRNA levels were determined by a reverse-transcription polymerase chain reaction. Inset: immunocytochemical detection of GHR on unstimulated DHD and RH cells: IgG (left) vs. GHR (right). Scale bar = 100 μm (B) Proliferation of DHD and RH cells in the presence or absence of GH (0.2 μg/mL) for the indicated periods of time. ***: *P* < .0005 vs. 0 h; **: *P* < .005 vs. 0 h; ns: nonsignificant vs. 0h.

To evaluate whether exogenous GH treatment could modulate cell proliferation in the tumor cell lines, we first confirmed GHR expression, both by immunodetection of the receptor and by real-time PCR. As previously reported [23,24], we showed that GH treatment regulates the expression of its own receptor in a tissue-dependent manner (Fig 2A). However, the cells treated with physiological GH doses showed no significant differences in their proliferation rates, as indicated by proliferation curves (Fig 2B).

### Immunohistochemical detection of GHR in the ileum and tumor is not affected by GH treatment

**Fig 3:**
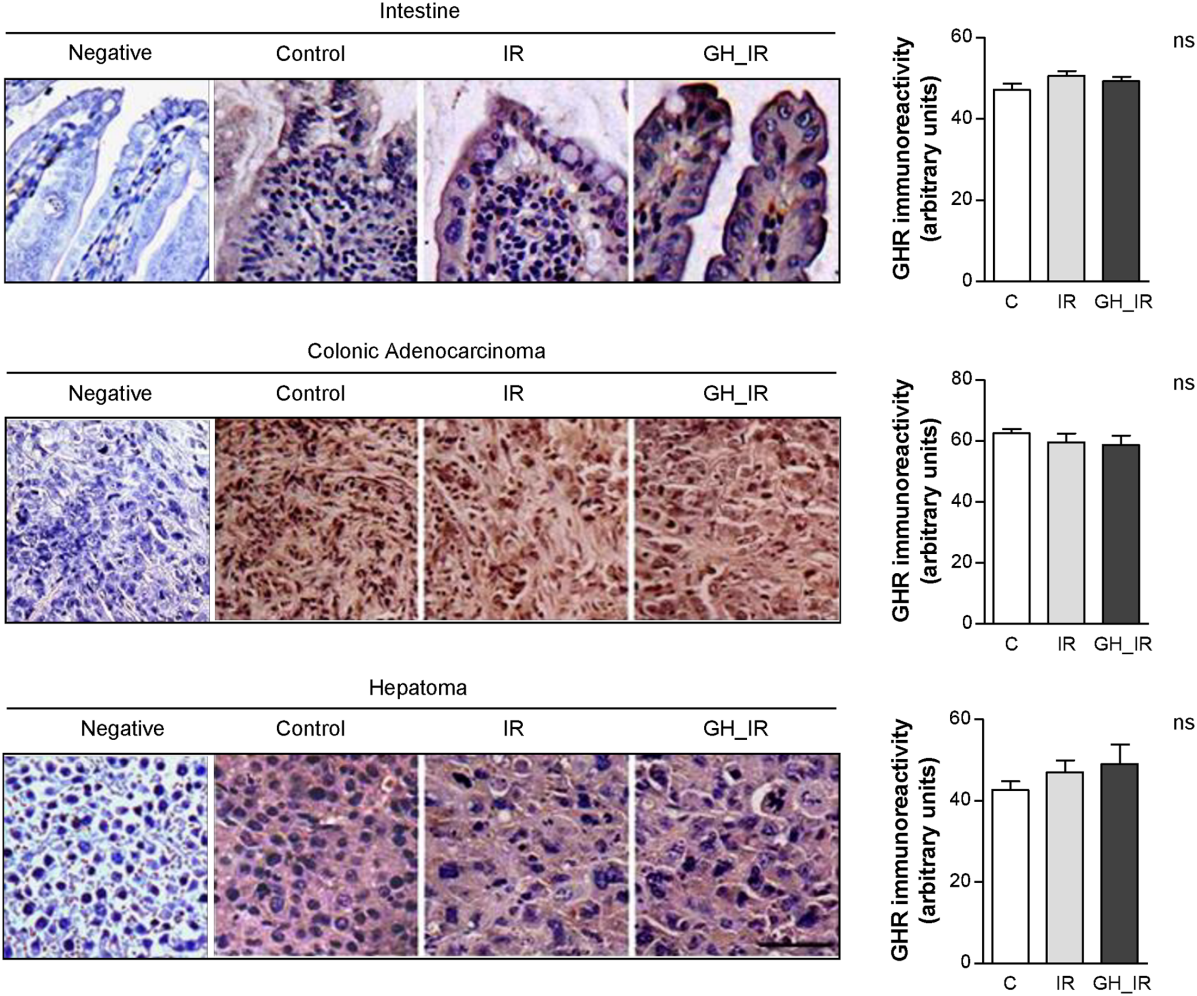
Immunohistochemical detection of GH receptors in the intestine and tumor tissue. Representative immunohistochemical staining with anti-GHR in the ileum and tumor tissue of the control, irradiated (IR), and GH-treated irradiated animals (GH_IR). GHR expression was quantified and expressed as arbitrary units using a semi-quantitative scoring system as described in the Materials and Methods section. ns: nonsignificant. Scale bar = 100 μm.

GH treatment is able to modulate GHR expression, but whether this effect persisted after radiotherapy was yet to be determined. Thus, we performed immunohistochemical detection of GHR on paraffin-embedded tissues, and GHR expression was quantified by using a semi-quantitative scoring system. In agreement with our *in vitro* results, at late time points, the semi-quantitative analyses indicated that there were no differences in GHR expression due to radiotherapy or GH treatment in the ileum or in either type of tumor (Fig 3).

### In the GH-treated animals, the small intestine epithelium is protected against radiation-induced injury, and caspase-3 activity is reduced in the intestine but not in the tumors

**Fig 4:**
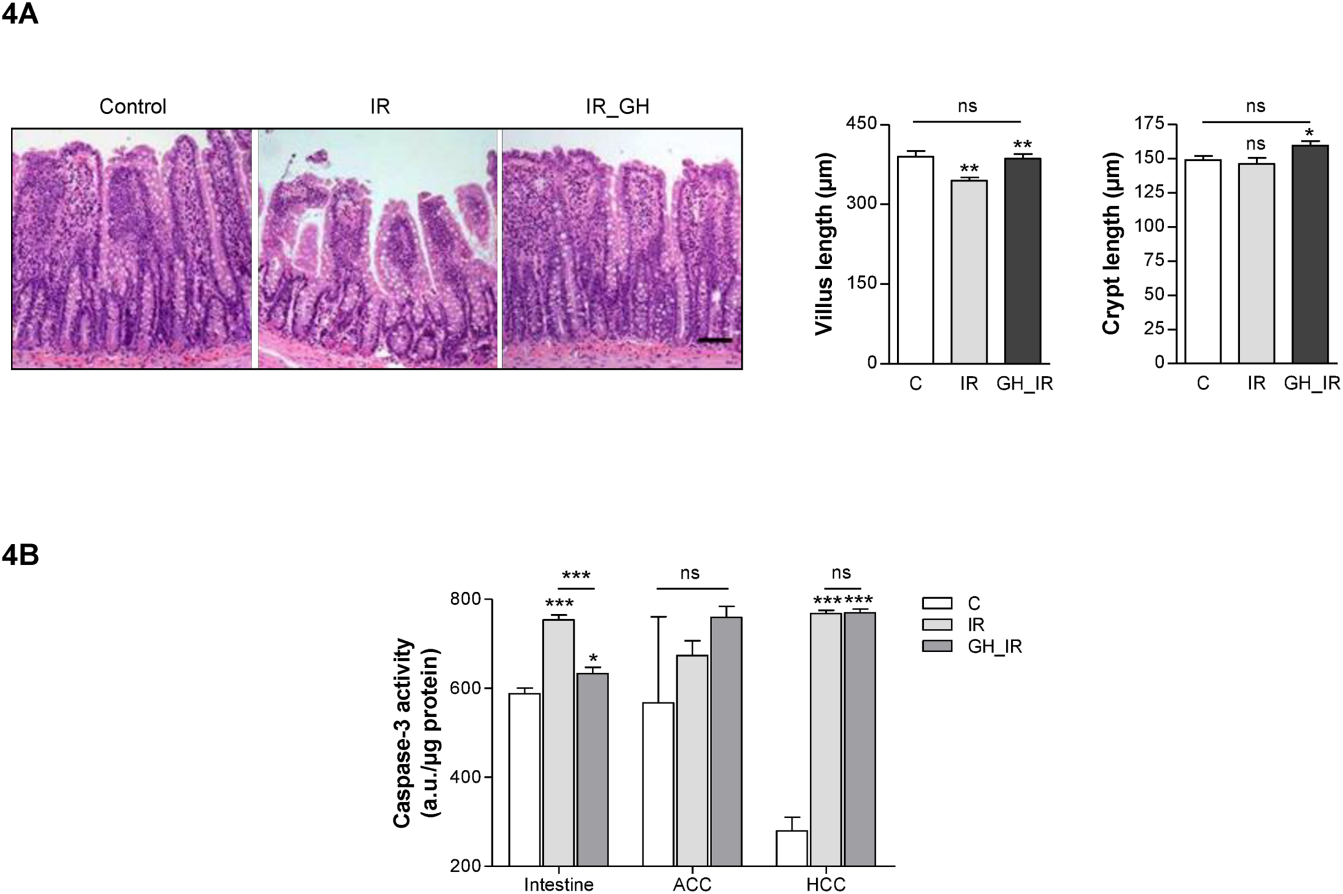
Influence of GH on radiation-induced tissue injury. (A) GH effects on ileum mucosal thickness. Representative hematoxylin-eosin sections of ileal mucosa from the control (C), irradiated (IR), or GH treated and irradiated (GH_IR) animals. Average values of villus and crypt length of ileum samples. **: *P* < .005 vs. control; *: *P* < .05 vs. control; ns: nonsignificant. (B) Apoptosis detection by caspase-3 activity. The role of GH in the prevention of radiotherapy-induced apoptosis was assessed by measuring the caspase-3 activity in cell lysates obtained from ileal mucosa and tumor tissue from the irradiated and the irradiated and GH-treated animals. ***: *P* < .0005 vs C; ***: *P* < .0005 IR vs GH_IR in intestine; *: *P* < .05 vs C; ns: nonsignificant.

Radiotherapy produces significant damage in the intestine. To analyze the effect of GH on this induced injury, we measured the lengths of the villi and crypts in the ileum. As expected, radiotherapy significantly decreased villi length in the IR group (344.8±6.1) compared with the control group (389.6±11.07). This effect was blocked by GH, and the epithelium of the GH-treated animals was protected against the typical changes induced by radiation in the bowel tissues: the GH_IR group had a normal villus length (386.2±8.7), significantly higher compared with the IR group, and GH treatment also produced a significant increase in the GH_IR crypts compared with the control and IR groups (Fig 4A).

Given radiation-induced apoptosis is one of the primary agents of intestinal injury, we evaluated the caspase-3 activity to verify whether GH treatment could have a protective effect on intestinal apoptosis, resulting in an improvement in the intestinal tissue. As shown in Fig 4B, GH treatment significantly reduced caspase-3 activity in the intestinal epithelium (IR: 753.5±11.1 compared with GH_IR: 633.3±14.07) but, interestingly, it did not diminish apoptotic activity in the tumors (ACC: IR: 673.7±33 compared with GH_IR: 759.2±25; HCC: IR: 767.5±7.5 compared with GH_IR: 769.6±8.4).

### GH treatment did not reduce radiotherapy-induced apoptosis in tumors or in cancer cell lines

**Fig 5:**
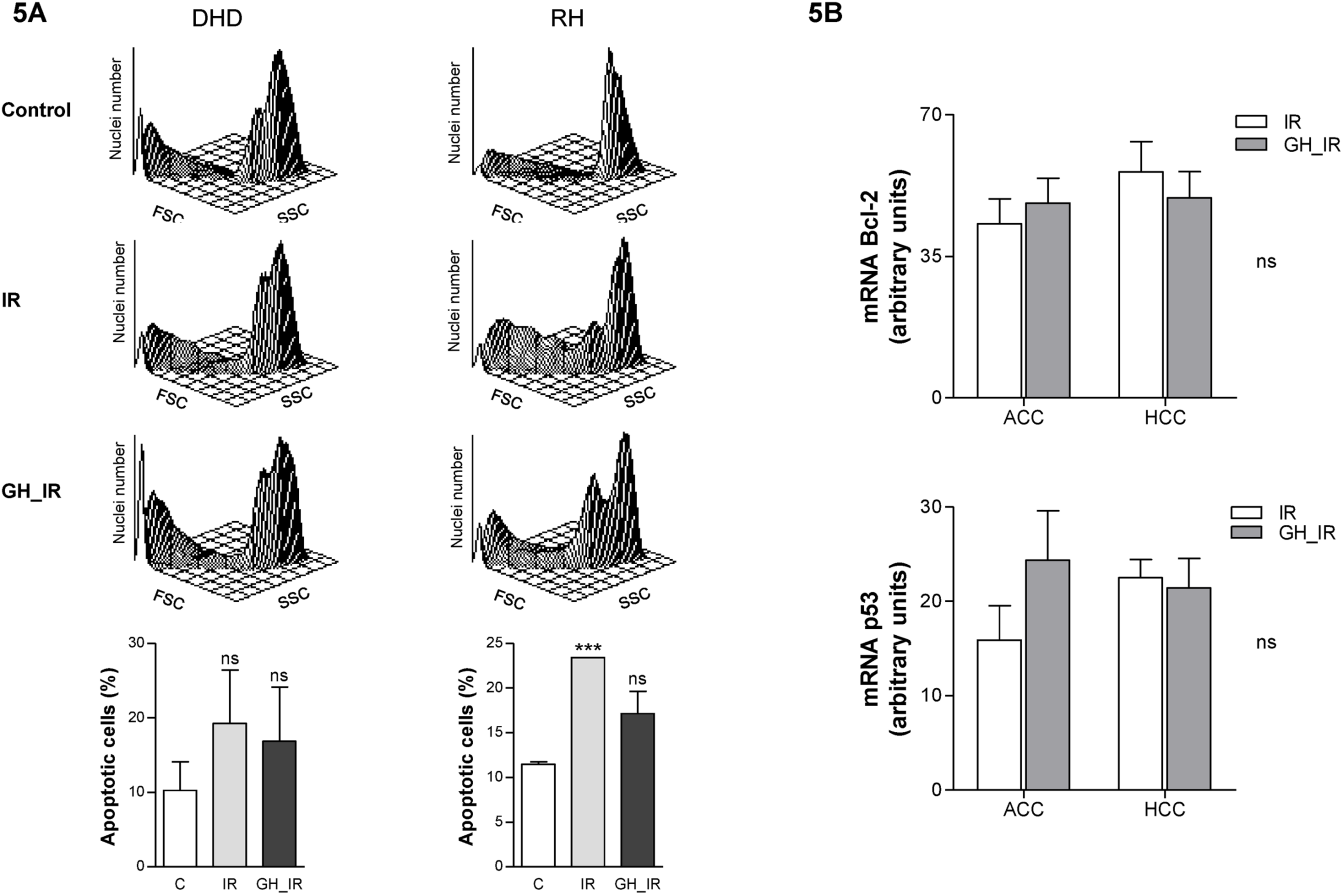
GH does not impair radiotherapy-induced apoptosis in tumor cells. (A) Flow cytometry analysis of the apoptotic population in DHD and RH cells, irradiated and treated or not with GH. Flow cytometry traces representative of the control and the irradiated cells treated with saline or 0.2 μg/mL of GH, and harvested at 24 h after radiation. Lower panel: quantification of apoptotic cells. ***: *P* < .0005 vs. C; ns: nonsignificant. (B) Analysis of Bcl-2 and p53 mRNA levels in colonic adenocarcinoma and hepatocarcinoma biopsies obtained from the irradiated animals. Gene expression was analyzed by reverse-transcription polymerase chain reaction. ns: nonsignificant.

In view of the caspase-3 results, and to further investigate the effect of GH on the apoptotic effects of radiotherapy in tumors, we analyzed apoptosis in the DHD and RH cell lines, both of which were pretreated with GH and radiated *in vitro*, using flow cytometry. As expected, the apoptotic population increased significantly after radiation, but it was not affected by GH treatment (Fig 5A), regardless of dose (data not shown).

As previously described, p53 and Bcl-2 play a central role in the mechanism of apoptosis-induced radiation toxicity [25,26]. We analyzed their mRNA levels in the adenocarcinoma and hepatoma biopsies obtained from the animals to evaluate whether GH was impairing the therapeutic effect of radiation on the tumors. In agreement with the caspase-3 activity shown in Fig 4B, there was no significant difference in the expression of the apoptosis-related genes after GH administration, suggesting that GH does not alter radiotherapy-induced tumor apoptosis (Fig 5B).

### GH does not affect cancer cell survival after irradiation

**Fig 6:**
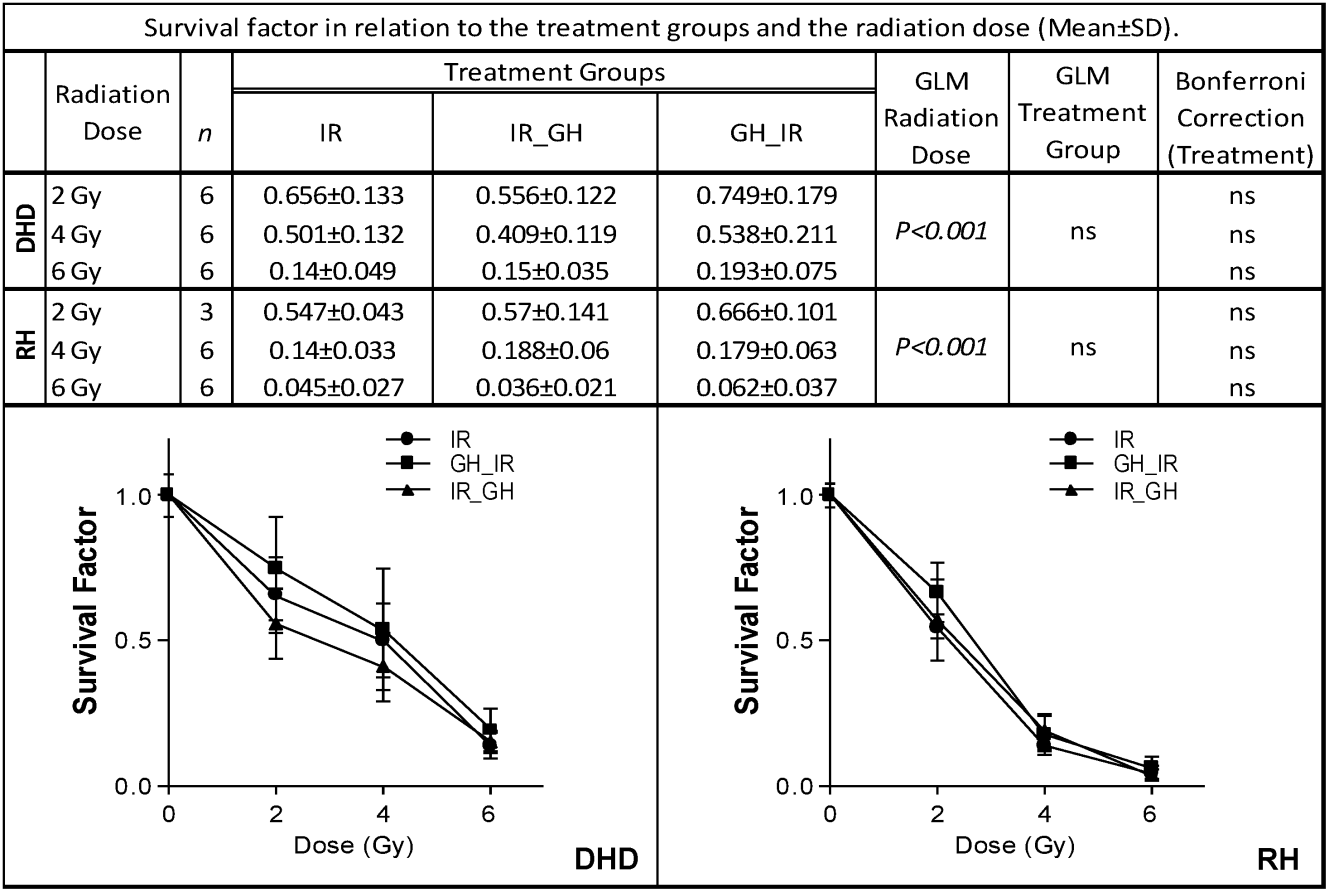
GH does not affect cancer cell survival after irradiation. Clonogenic surviving fraction of DHD and RH carcinoma cells following treatment with radiation alone (IR) or radiation with GH administered prior (GH_IR) or following the irradiation (IR_GH). Upper panel: statistical analysis. Lower panel: Survival curves of DHD (left) and RH (right) cells after irradiation. As a result, GH does not affect clonogenic survival in irradiated tumor cells regardless of the treatment received (*P* = ns). The surviving fraction of cells was normalized by the occurrence of cell death in the group treated with 0 Gy radiation (nonirradiated and unstimulated). GLM: General Linear Model; ns: nonsignificant.

Our data suggest that, at physiological doses, GH does not increase tumor cell proliferation, but there is no evidence regarding whether GH treatment modifies the survival of cancer cells following irradiation. To evaluate the possible effect of GH, we performed a clonogenic survival assay. After the statistical analysis (Fig 6), it was observed that survival factor (SF) is clearly conditioned by the radiation dose the cells are subjected to (both DHD and RH). As expected, SF decreases significantly according to the increase in radiation dose (*P* < .001). However, when SF was analyzed in relation to the treatment received, no statistically significant differences were found in either DHD or RH (*P* = ns). A detailed analysis of both cell types showed that SF remained stable regardless of the treatment received (Bonferroni correction). Therefore, the SF of the GH-treated groups (before or after RT) does not differ from the irradiated cells despite the radiation dose received, ruling out the possibility of a radiosensitizing effect by GH, given there were no significant differences in survivability between cells irradiated alone or in combination with GH.

### GH effect on cell proliferation

**Fig 7:**
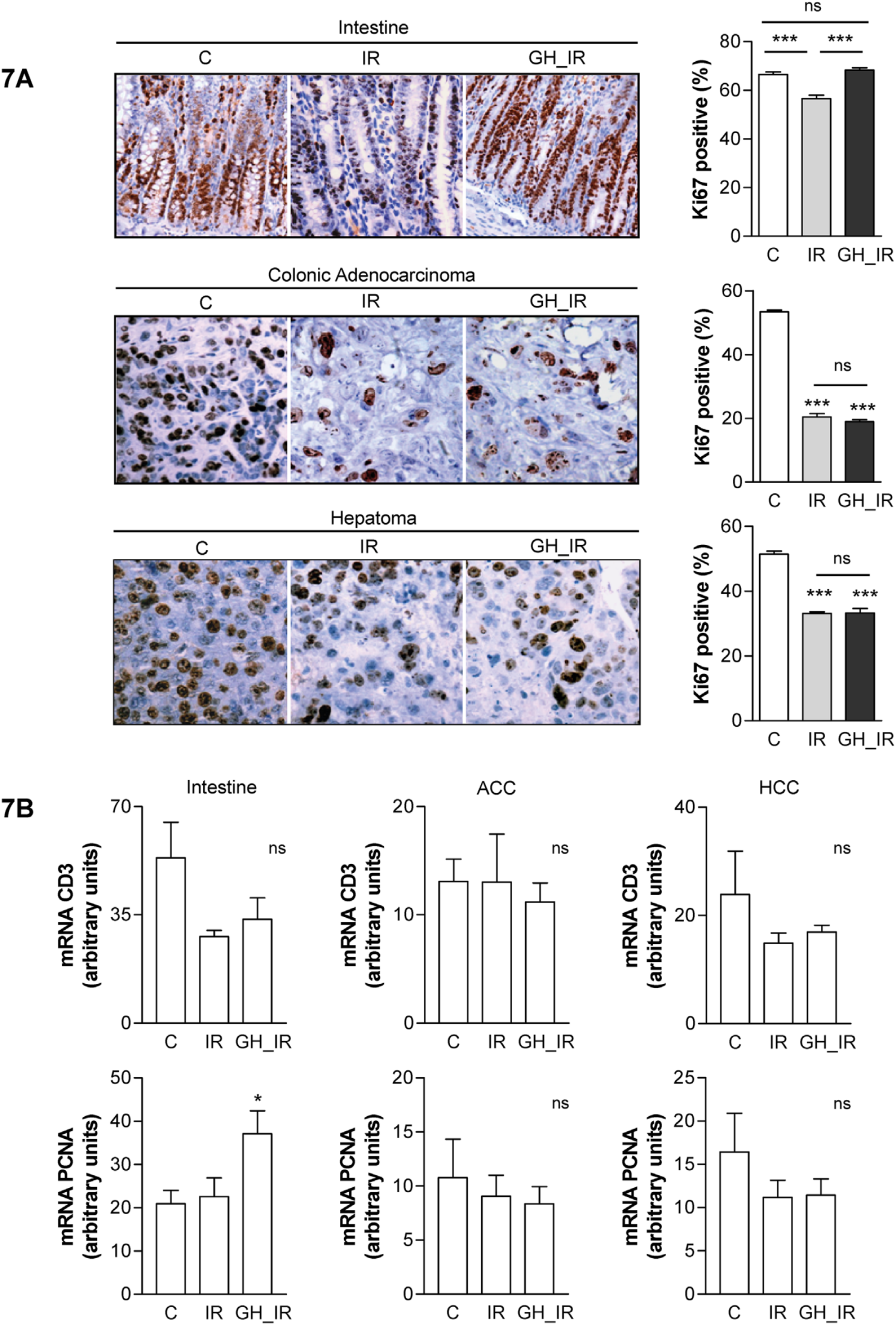
GH treatment interferes with cell-cycle progression after irradiation in the intestine but not in tumors. (A) Representative images of immunostaining for the proliferating nuclear antigen Ki-67, which is expressed in replicating cells throughout the cell cycle, in ileal and tumor tissue. Positive Ki-67 nuclei were counted in 10 consecutive fields from each section (magnification, 200×). Intestine: ***: *P* < .0005 IR vs. C and IR vs GH_IR; Tumor: ***: *P* < .0005 vs. C; ns: nonsignificant. (B) Expression of cyclin D3 and PCNA mRNA in the intestine and tumors of the animals. *: *P* < .05 vs. control; ns: nonsignificant.

To evaluate whether GH treatment was important for intestinal epithelium regeneration and for tumor progression, we analyzed the potential proliferative effect of GH on the irradiated ileum and tumors by Ki67 immunostaining. As shown in Fig 7A, radiotherapy significantly decreased crypt cell proliferation in the intestine of the irradiated group (IR: 56%±4%) compared with the control group (C: 66%±2%). GH treatment prevented this effect, and Ki67 staining showed similar levels of cell proliferation in the GH_IR group (68%±3%) as in the control group, together with a significantly increased cell proliferation compared with the IR group.

Although radiotherapy significantly decreased tumor proliferation both in colonic adenocarcinoma (IR: 20%±3%) and in hepatoma (IR: 33%±1%), GH treatment did not increase tumor proliferation after radiotherapy in either (ACC GH_IR: 19%±2%; HCC GH_IR: 33%±3%).

To confirm the Ki67 labeling, we analyzed the proliferation markers cyclin D3 and PCNA, both of which are involved in the proliferative effects reported for GH [27,28]. Consistent with the proliferation data obtained from Ki67, there was a slight increase in the intestinal tissue of the mRNA levels of both genes, but there were no differences in the expression of these markers after GH treatment in either of the tumors (Fig 7B).

### FDG uptake in GH-treated animals increases in the intestine but not in tumor tissue

We assessed the *in vivo* response to radiotherapy in the small intestine and tumors by employing PET quantification of FDG uptake. FDG uptake was measured in basal conditions, 2 weeks after tumor cell inoculation without any treatment, and 3 days after the final irradiation (Fig 1). Liver uptake was used for normalization of both the small intestine and the tumors.

**Fig 8:**
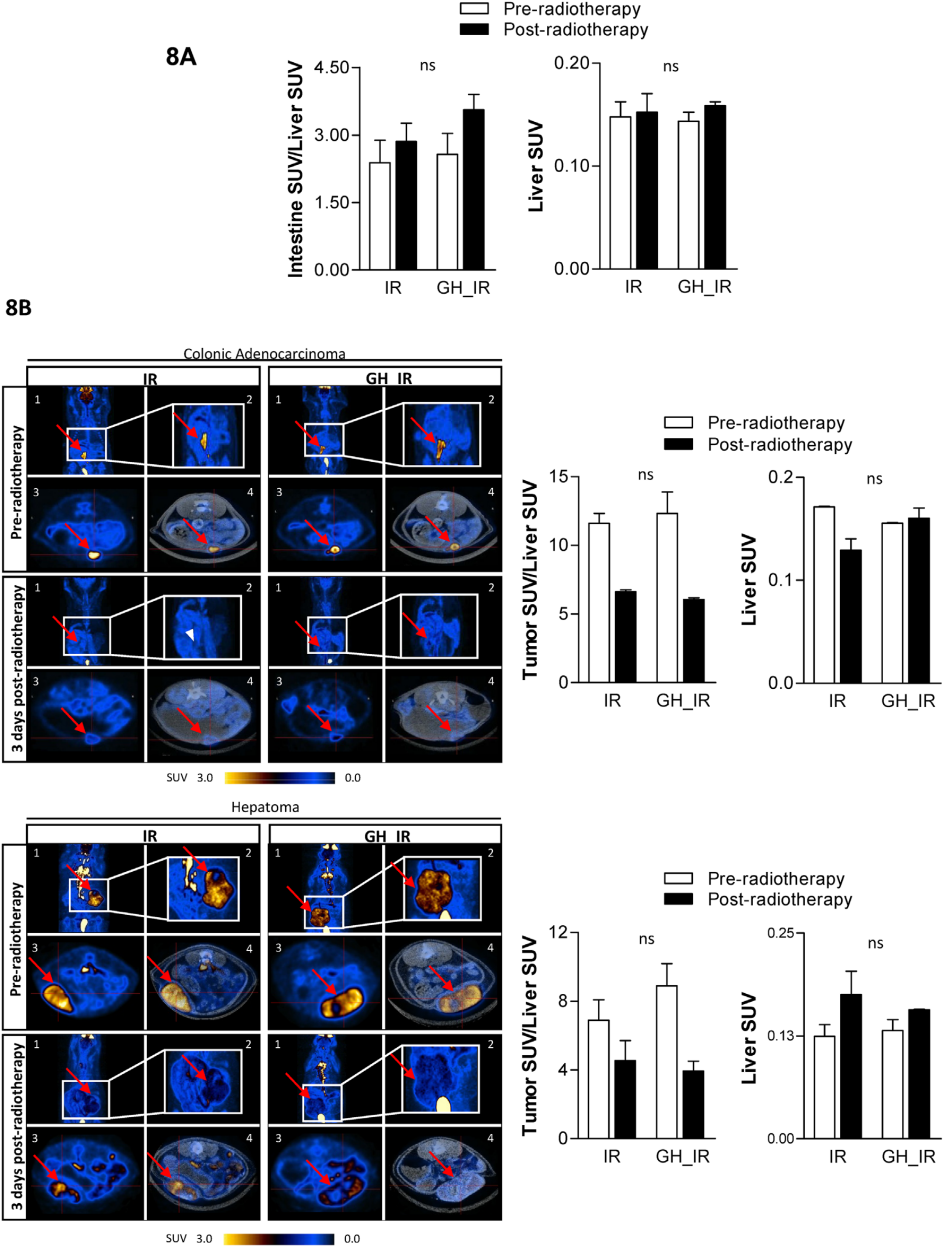
Effects of GH on tumor and intestine response to radiotherapy evaluated by FDG PET/CT. Quantification of FDG uptake, expressed as UV, before radiotherapy (white bars) and 3 days after radiotherapy (black bars) of the irradiated (IR) and GH-treated and irradiated animals (GH_IR). The data were normalized with normal liver SUV values. (A) Small intestine (B) Colonic adenocarcinoma and hepatoma: (1) Three-dimensional reconstruction of the coronal PET image of the complete animal with a window (2) showing an enlarged abdominal zone, (3) transaxial slices of PET, and (4) PET-CT fusion images. The red arrowheads point to the tumor. Lower panel: Quantification of FDG uptake in tumor tissue expressed as SUV and normalized with their respective liver values. ns: nonsignificant.

After radiation, intestinal FDG uptake was augmented in all the animals, but there was a more intense increase in the GH-treated group compared with the non-GH-treated group (almost a 2-fold increase: 38.52% versus 19.75%, respectively). This result suggests a higher metabolic rate in the intestine of the GH-treated animals, which could be compatible with a higher proliferative activity (Fig 8A). However, it is important to take into account that the inflammatory component due to irradiation cannot be ruled out and might contribute to the observed FDG activity.

Regarding tumor tissue, both ACC and HCC showed a suitable response to radiotherapy, as shown by the marked effect on the reduction in FDG uptake. However, unlike that observed in the irradiated ileum, the GH treatment did not affect FDG uptake in either tumor. After irradiation, tumors from the GH-treated animals exhibited reduced metabolic activity compared with the nontreated animals (Fig 8B). In ACC, after irradiation, SUV values decreased 42.94% in the IR group versus 50.91% in the GH_IR group; in HCC, FDG uptake clearly decreased after radiotherapy in the animals treated either with or without GH, and SUV values decreased 34.20% in the IR group versus 55.71% in the GH_IR group.

These results confirmed by *in vivo* imaging that the metabolic response of both kinds of tumors to radiotherapy was not modified by GH treatment, indicating that GH did not increase the cell viability of either tumor type after radiotherapy.

## Discussion

In the present study, we show that in animal models bearing tumors, the exogenous administration of GH concomitant with radiation preserves the intestine against acute radiation-induced injury, yet exerts its action without protecting ACC or HCC, while maintaining the antitumor effect of radiotherapy. Along these lines, we have previously reported the proliferative effect of GH on the intestine and its protective action against radiotherapy and methotrexate-induced injury [8,9,29–31].

Our data show that GH treatment notably improves the proliferative and regenerative potential of the intestinal epithelium, as assessed by crypt and villi measurements as well as Ki67 labeling and PCNA and cyclin D3 expression; at the same time, we have shown a drop in caspase-3 activity in the intestinal epithelium after GH treatment. These data were consistent with previous studies of our group, in which GH was effective in preventing apoptosis induced by radiation and chemotherapy [30, 32]. This reduced apoptosis together with its augmented proliferative activity could lead to an increase in the number of stem cells before radiation, improving the probability of their survival after radiotherapy or producing faster intestinal mucosa regeneration [7,8]. Apoptosis is known to be one of the central mechanisms of radiation toxicity, and those cells primarily affected are the cells of the rapidly dividing intestinal epithelium, particularly crypt stem cells [33–35]. Therefore, a key factor in preventing the toxic effects of radiation in the intestine would be the survival and proliferation of these stem cells after radiotherapy, given a single stem cell can regenerate the crypt in the first 48–72 h after irradiation [35]. GH has been proposed as a protective factor for the intestine because it is able to exert both antiapoptotic and proliferative effects on the intestine of irradiated animals, improving mucosal trophism in irradiated rats and mobilizing endothelial progenitor cells in healthy adults [12,36].

The striking result was that, while protecting the intestine, GH did not impair the antitumor effect of the radiation. Despite its proven mitotic potential in nontumor cells, after GH administration *in vitro*, the DHD and RH cells did not significantly increase their proliferation. We have verified that both the cell lines and the tumors were positive for GHR, and were able to regulate its expression, ruling out the possibility that the lack of GH effects on those tumors was due to the absence of GHR. Moreover, when we analyzed the irradiated tumors, GH treatment did not improve Ki67 labeling after irradiation, and this lack of proliferative effect was assessed by the expression of cyclin D3 and PCNA. Furthermore, the apoptotic effect of radiation was maintained after GH administration, as shown by p53 and Bcl2 expression, and by the quantification of apoptotic cells by flow cytometry. Similarly, the clonogenic survival assay showed that GH treatment (before or following RT) did not modify the survival capacity of tumor cells after irradiation, nor did it have a radiosensitizing effect. Thus, GH appears to be a growth factor that behaves differently depending on the tissue studied. This dual action is not exclusive to GH: similar to our findings, KGF, a member of the fibroblast growth factor family, has proven mitotic activity, improving crypt cell survival in irradiated animals [37], while not enhancing the growth of various cancer cells.

Data obtained from the FDG PET/CT permits the *in vivo* assessment of the early tumor response to radiotherapy [38], as is routinely performed in the clinic. Specifically, the reduction in FDG uptake is a sensitive marker of the reduction in viable tumor tissue used for the evaluation of the tumoricidal effect of radiotherapy. In mice, a proliferative response occurs within 48–72 h after radiotherapy [39], providing a time window suitable for evaluating the effect of proliferation stimulators [40], which is why we assessed the GH effects 3 days after radiotherapy. Our results indicate that in the small intestine, FDG uptake after radiotherapy increased almost twice as much in the GH_IR group as in the IR group. Concurrently, FDG uptake decreased in the ACC and HCC tumors after radiotherapy, indicating that radiotherapy was effective, and the early tumor response to fractionated radiotherapy was suitable [38]. However, RT induces a strong inflammatory reaction that also contributes to the signal, so it is important to take into account that the source of the signal cannot be attributed exclusively to the metabolic status of the cell, and the inflammatory component must also be considered. Interestingly, FDG uptake decreased both in the GH- and the non-GH-treated animals, and the ratio was very similar in the two groups, involving a response not impaired by GH treatment. Thus, the *in vivo* metabolic status of the tumors does not appear to be improved by GH administration, correlating with our findings on proliferation and apoptotic tumor rates.

The absence of a pro-tumor effect of GH in animal models was suggested by Brennan et al., concluding that GH stimulates liver protein synthesis without changing tumor growth, protein synthesis, or host composition in a rat sarcoma model after 2 weeks of treatment [15]. Moreover, in an animal model of pulmonary metastasis in rats, GH treatment not only did not stimulate tumor primary growth but also inhibited its metastasis [41]. In studies that developed human tumors in nude mice, a pro-oncogenic effect was found: in a wide preclinical study of 20 human tumor models established in nude mice under continuous exposure to GH, no evidence for tumor growth stimulation was found in any of the tumors studied [42], and no effect was found when the proliferation of human gastric carcinoma cells was studied *in vivo* or *in vitro* [43,44].

Regardless of the promising results obtained in animals, given the proven mitotic and anabolic properties of GH [24], the use of GH in patients is a matter of great concern because it might increase the risk of future cancer. Since the development and approval of synthetic human GH (hGH) in 1985, the FDA has authorized it only for a few specific uses in children and adults: In children, for treating short stature of unknown cause as well as poor growth due to Turner's syndrome, Prader-Willi syndrome, chronic renal insufficiency, GH insufficiency, or children born small for gestational age. In adults, the use of GH is approved only in short bowel syndrome, GH deficiency due to pituitary tumors, or muscle-wasting disease associated with HIV/AIDS [45]. Other uses, such as for cachexia or treatment of surgical patients, are still under study despite favorable evidence [14,41,46].

However, the primary conflict likely centers on the treatment of oncological patients under radiotherapy, given on one side the reported benefits of GH in preventing radiation-related healthy tissue injury, and on the other its pro-proliferative potential [13,47,48]. In recent years, an increasing amount of data from long-term patient surveillance and meta-analysis studies suggest the safety of low-dose GH treatment. A report studying the risk of recurrence and second neoplasms in survivors of childhood cancer treated with GH concludes that GH therapy does not appear to increase the risk [49]. The same conclusion was obtained in a study of 58,603 patients from the Pfizer International Growth Database, in which no association was found between GH treatment and an increase in the incidence of cancer [50], nor was an association found in a cohort of 6840 GH-treated adult hypopituitary patients [51]. Reassuringly, the conclusions extracted from studies around the world that include considerable follow-up data indicate there is no clear evidence from clinical practice that GH treatment has a causal relationship with tumor occurrence or recurrence [52,53]. A 2012 study conducted in France has rekindled the controversy, reporting that patients with idiopathic GH deficiency and idiopathic or gestational short stature who were treated with recombinant hGH during childhood were at a small increased risk of death when compared with individuals in the general French population [54]. Interestingly, the study states that the risk of death was increased when doses of GH that are higher than normally prescribed were used. Although the FDA is currently reviewing all available information, its latest recommendation for healthcare professionals is the “FDA believes the benefits of recombinant growth hormone continue to outweigh its potential risks” [45].

Although the antiapoptotic, mitogenic, and anabolic effect of GH is undeniable, however, the exact mechanism by which tumor cells are unprotected remains unknown; thus, the threat of a potential adverse effect from GH, including increased future cancer risk or tumor recurrence, precludes its clinical use in oncological patients. According to Lobie and Devesa [55,56], the key could be not only in the dose, but in the nature of GH. These authors stated that, as opposed to autocrine GH, exogenous GH administration does not appear to be related to oncogenesis because the oncogenic properties of GH are exclusive of the locally produced hormone. The exogenous hormone, with pulsatile changes in its concentration, would mimic the effect of the endocrine-delivered GH that does not induce oncogenic transformation [57,58]. Moreover, microarray analysis has confirmed differential regulation of a subset of more than 300 genes that are specifically regulated by autocrine production of hGH in mammary carcinoma cells and not by exogenously added hGH [59]. Thus, just as exogenous GH induces a transient activation of P44/42 MAP kinase, autocrine GH induces a sustained activation of the mitogenic pathway, at least for 48 h, triggering a potent oncogenic effect.

The effect produced by IGF-1 is significantly different. IGF-1 is important for both the regulation of normal physiology and a number of pathological states, including cancer, and it has been shown to play roles in the promotion of cell proliferation and in the inhibition of apoptosis [60]. Because IGF-1 is primarily secreted by the liver as a result of stimulation by GH, it is widely held that the GH/IGF-1 axis plays a role in the development and progression of cancer, although the exact pathophysiology of neoplastic transformation is not clear, and the role of GH has not been determined. However, IGF-1’s induction of anomalous proliferation apparently occurs only when elevated serum concentrations are found; thus, GH administration must be adjusted to doses that maintain circulating IGF-1 levels less than 2 standard deviations (SD) below the age-matched mean [56,61]. In this sense, treatment with ghrelin, the natural endogenous ligand for GH secretagogue receptors, increases GH levels but has a relative lack of effect on IGF-1 plasma levels [62], supporting the independent actions of both factors. Moreover, ghrelin administration mimics some of the results that we obtained with GH treatment: it has proven to be effective in mitigating intestinal injury after irradiation in rats [48], promoting mucosal proliferation, reducing apoptosis in the elemental diet-induced hypotrophic intestine [62], and in the chemoprevention of inflammation-associated colorectal carcinogenesis, but without tumor-promoting effects [46].

In conclusion, despite the proliferative effect of GH, our data suggest that short-term administration of GH concomitant with radiotherapy exerts a dual action, preventing radiation injury to the intestine while not protecting the tumor, preserving the therapeutic potential of the treatment. The promising role of ghrelin and the increasing evidence indicating a differentiating activity between IGF-1 and GH, could be the missing link to explain the dual action of GH, and opens the door for future investigations. However, it is important to emphasize that our data derive from a preliminary animal study, and any question about the potential clinical use of GH is far beyond the scope of the present manuscript. A longer follow-up and additional studies are needed to determine whether this effect can be extrapolated to other tumors, and a lack of pro-oncogenic action of GH on healthy tissue should be fully confirmed.

## References

[1] Shadad AK, Sullivan FJ, Martin JD, Egan LJ. Gastrointestinal radiation injury: Prevention and treatment. World J Gastroenterol. 2013;19: 199–208. 10.3748/wjg.v19.i2.199 23345942 PMC3547575

[2] Waselenko JK, MacVittie TJ, Blakely WF, Pesik N, Wiley AL, Dickerson WE, et al National Stockpile Radiation Working Group. Medical management of the acute radiation syndrome: recommendations of the Strategic National Stockpile Radiation Working Group. Ann Intern Med. 2004;140: 1037–1051. 15197022 10.7326/0003-4819-140-12-200406150-00015

[3] Stacey R, Green JT. Radiation-induced small bowel disease: latest developments and clinical guidance. Ther Adv Chronic Dis. 2014;5: 15–29. 10.1177/2040622313510730 24381725 PMC3871275

[4] Harb AH, Abou Fadel C, Sharara AI. Radiation Enteritis. Curr Gastroenterol Rep. 2014;16: 383 10.1007/s11894-014-0383-3 24604730

[5] Denham JW, Hauer-Jensen M. Radiation induced bowel injury: a neglected problem. Lancet. 2013;382: 2046–2047. 10.1016/S0140-6736(13)61946-7 24067489

[6] Gomez de Segura IA, Valderrabano S, Vazquez I, Vallejo-Cremades MT, Gomez-Garcia L, Sanchez M, et al Protective effects of dietary enrichment with docosahexaenoic acid plus protein in 5-fluorouracil-induced intestinal injury in the rat. Eur J Gastroenterol Hepatol. 2004;16: 479–485. 15097041 10.1097/00042737-200405000-00008

[7] Booth D, Potten CS. Protection against mucosal injury by growth factors and cytokines. J Natl Cancer Inst Monogr. 2001;29: 16–20. 11694560 10.1093/oxfordjournals.jncimonographs.a003433

[8] Vazquez I, Gomez de Segura IA, Grande AG, Escribano A, Gonzalez-Gancedo P, Gomez A, et al Protective effect of enriched diet plus growth hormone administration on radiation-induced intestinal injury and on its evolutionary pattern in the rat. Dig Dis Sci. 1999;44: 2350–2358. 10573386 10.1023/a:1026637611298

[9] Posadas SJ, Largo C, Merino JJ, Elvira M, Gonzalez G, Caz V, et al Growth hormone upregulates intestinal trefoil factor expression in the ileum of rats after radiation. Exp Biol Med. 2011:236: 205–211. 10.1258/ebm.2010.009358 21321317

[10] Beck PL, Wong JF, Li Y, Swaminathan S, Xavier RJ, Devaney KL, et al Chemotherapy- and radiotherapy-induced intestinal damage is regulated by intestinal trefoil factor. Gastroenterology. 2004;126: 796–808. 14988834 10.1053/j.gastro.2003.12.004

[11] Carlo-Stella C, Di Nicola M, Milani R, Longoni P, Milanesi M, Bifulco C, et al Age- and irradiation-associated loss of bone marrow hematopoietic function in mice is reversed by recombinant human growth hormone. Exp Hematol. 2004;32: 171–178. 15102478 10.1016/j.exphem.2003.11.007

[12] Devin JK, Vaughan DE, Blevins LS, Chen Q, Covington J, Verity DK, et al Low-dose growth hormone administration mobilizes endothelial progenitor cells in healthy adults. Growth Horm IGF Res. 2008;18: 253–63. 10.1016/j.ghir.2007.11.001 18166495

[13] Tekin SB, Ertekin MV, Erdogan F, Sezen O, Karslioglu I, Gepdiremen A, et al Is growth hormone a radioprotective agent? J Eur Acad Dermatol Venereol. 2006;20: 293–298. 16503890 10.1111/j.1468-3083.2006.01454.x

[14] Trobec K, von Haehling S, Anker SD, Lainscak M. Growth hormone, insulin-like growth factor 1, and insulin signaling-a pharmacological target in body wasting and cachexia. J Cachexia Sarcopenia Muscle. 2011;2: 191–200. 22207907 10.1007/s13539-011-0043-5 PMC3222822

[15] Wolf RF, Ng B, Weksler B, Burt M, Brennan MF. Effect of growth hormone on tumor and host in an animal model. Ann Surg Oncol. 1994;1: 314–20. 7850530 10.1007/BF02303570

[16] Costoya JA, Rios R, Garcia-Barros M, Gallego R, Garcia-Caballero T, Señaris R, et al Role of growth hormone receptor in HL-60 cell survival. Mol Cell Biol Res Commun. 2000;4: 26–31. 11152624 10.1006/mcbr.2000.0252

[17] Zhu Z, Mukhina S, Zhu T, Mertani HC, Lee KO, Lobie PE. P44/42 MAP kinase dependent regulation of catalase by autocrine human growth hormone protects human mammary carcinoma cells from oxidative stress-induced apoptosis. Oncogene. 2005;24: 3774–3785. 15782123 10.1038/sj.onc.1208541

[18] Lobie PE, Breipohl W, Waters MJ. Growth hormone receptor expression in the rat gastrointestinal tract. Endocrinology. 1990;126: 299–306. 2293990 10.1210/endo-126-1-299

[19] Santamaria M, Pardo-Saganta A, Alvarez-Asiain L, Di Scala M, Qian C, Prieto J, et al Nuclear α1-antichymotrypsin promotes chromatin condensation and inhibits proliferation of human hepatocellular carcinoma cells. Gastroenterology. 2013;144: 818–828. 10.1053/j.gastro.2012.12.029 23295442

[20] Gonzalez-Rodriguez A, Escribano O, Alba J, Rondinone CM, Benito M, Valverde AM. Levels of protein tyrosine phosphatase 1B determine susceptibility to apoptosis in serum-deprived hepatocytes. J Cell Physiol. 2007;212: 76–88. 17323378 10.1002/jcp.21004

[21] Riccardi C, Nicoletti I. Analysis of Apoptosis by propidium iodide staining and flow cytometry. Nature Protocols. 2006;1: 1458–1461. 17406435 10.1038/nprot.2006.238

[22] Louis E, Ancion G, Colard A, Spote V, Belaiche J, Hustinx R. Noninvasive assessment of Crohn’s disease intestinal lesions with 18F-FDG PET/CT. J Nucl Med. 2007;48: 1053–1059. 17574978 10.2967/jnumed.107.040436

[23] Bennett WL, Ji S, Messina JL. Insulin regulation of growth hormone receptor gene expression. Evidence for a transcriptional mechanism of down-regulation in rat hepatoma cells. Mol Cell Endocrinol. 2007;274: 53–59. 17658679 10.1016/j.mce.2007.05.020

[24] Jiang J, Wan Y, Wang X, Xu J, Harris JM, Lobie PE, et al Inhibitory GH receptor extracellular domain monoclonal antibodies: three-dimensional epitope mapping. Endocrinology. 2001;152: 4777–4788. 10.1210/en.2011-1336 PMC3230063 21990310

[25] Liang H, Zhan HJ, Wang BG, Pan Y, Hao XS. Change in expression of apoptosis genes after hyperthermia, chemotherapy and radiotherapy in human colon cancer transplanted into nude mice. World J Gastroenterol. 2007;13: 4365–4371. 17708613 10.3748/wjg.v13.i32.4365 PMC4250866

[26] Lee CL, Blum JM, Kirsch DG. Role of p53 in regulating tissue response to radiation by mechanisms independent of apoptosis. Transl Cancer Res. 2013;2: 412–421. 24466508 PMC3898670

[27] Jeay S, Sonenshein GE, Postel-Vinay MC, Kelly PA, Baixeras E. Growth hormone can act as a cytokine controlling survival and proliferation of immune cells: new insights into signaling pathways. Mol Cell Endocrinol. 2002;188: 1–7. 11911939 10.1016/s0303-7207(02)00014-x

[28] Gomez de Segura IA, Aguilera MJ, Codesal J, Codoceo R, De Miguel E. Comparative effects of growth hormone in large and small bowel resection in the rat. J Surg Res. 1996;62: 5–10. 8606509 10.1006/jsre.1996.0164

[29] Gomez de Segura IA, Prieto I, Grande AG, García P, Guerra A, Mendez J, et al Growth hormone reduces mortality and bacterial translocation in irradiated rats. Acta Oncol. 1998;37: 179–85. 9636013 10.1080/028418698429748

[30] Morante J, Vallejo-Cremades MT, Gomez-Garcia L, Vazquez I, Gomez de Segura IA, Sanchez M, et al Differential action of growth hormone in irradiated tumoral and non tumoral intestinal tissue. Dig Dis Sci. 2003;48: 2159–2166. 14705822 10.1023/b:ddas.0000004520.71462.c9

[31] Ortega M, Gomez de Segura IA, Vazquez I, Lopez JM, de Guevara CL, de Miguel E. Effects of growth hormone plus a hyperproteic diet on methotrexate-induced injury in rat intestines. Acta Oncol. 2001;40: 615–621. 11669334 10.1080/028418601750444169

[32] Clavijo J, Gomez de Segura IA, Gomez-Garcia L, Vallejo-Cremades MT, Sanchez M, de Miguel E. Growth hormone protects the intestines but not the tumour from 5-fluorouracil toxicity in the short term in the rat. Eur J Gastroenterol Hepatol. 2004; 16: 75–82. 15095856 10.1097/00042737-200401000-00012

[33] Yu J. Intestinal stem cell injury and protection during cancer therapy. Transl Cancer Res. 2013;2: 384–396. 24683536 PMC3966653

[34] Potten CS, Booth C. The role of radiation-induced and spontaneous apoptosis in the homeostasis of the gastrointestinal epithelium: a brief review. Comp Biochem Physiol B Biochem Mol Biol. 1997;118: 473–8. 9467859 10.1016/s0305-0491(97)00219-8

[35] Potten CS. A comprehensive study of the radiobiological response of the murine (BDF1) small intestine. Int J Radiat Biol. 1990;58: 925–973. 1978853 10.1080/09553009014552281

[36] Raguso CA, Leverve X, Pichard C. Protective effects of recombinant growth hormone on intestinal mucosa in rats receiving abdominal radiotherapy. Clin Nutr. 2002;21: 487–490. 12468368 10.1054/clnu.2002.0579

[37] Ning S, Shui C, Khan WB, Benson W, Lacey DL, Knox SJ. Effects of keratinocyte growth factor on the proliferation and radiation survival of human squamous cell carcinoma cell lines *in vitro* and *in vivo* . Int J Radiat Oncol Biol Phys. 1998;40: 177–187. 9422575 10.1016/s0360-3016(97)00561-0

[38] Ichiya Y, Kuwabara Y, Otsuka M, Tahara T, Yoshikai T, Fukumura T, et al Assessment of response to cancer therapy using fluorine-18-fluorodeoxyglucose and positron emission tomography. J Nucl Med. 1991;32: 1655–1660. 1880564

[39] Potten CS, Owen G, Roberts SA. The temporal and spatial changes in cell proliferation within the irradiated crypts of the murine small intestine. Int J Radiat Biol. 1990;57: 185–199. 1967288 10.1080/09553009014550431

[40] Potten CS, Owen G, Hewitt D, Chadwick CA, Hendry H, Lord BI, et al Stimulation and inhibition of proliferation in the small intestinal crypts of the mouse after in vivo administration of growth factors. Gut. 1995;36: 864–873. 7615275 10.1136/gut.36.6.864 PMC1382624

[41] Torosian MH. Growth hormone and prostate cancer growth and metastasis in tumor-bearing animals. J Pediatr Endocrinol. 1993;6: 93–97. 8374696 10.1515/jpem.1993.6.1.93

[42] Fiebig HH, Dengler W, Hendriks HR. No evidence of tumor growth stimulation in human tumors in vitro following treatment with recombinant human growth hormone. Anticancer Drugs. 2000;11: 659–664. 11081460 10.1097/00001813-200009000-00011

[43] Liang DM, Chen JY, Zhang Y, Gan P, Lin J, Chen AB. Effects of recombinant human growth hormone on growth of human gastric carcinoma xenograft model in nude mice. World J Gastroenterol. 2006;12: 3810–3813. 16804963 10.3748/wjg.v12.i24.3810 PMC4087926

[44] Chen JY, Liang DM, Gan P, Zhang Y, Lin J. In vitro effects of recombinant human growth hormone on growth of human gastric cancer cell line BGC823 cells World J Gastroenterol. 2004;10: 1132–1136. 15069712 10.3748/wjg.v10.i8.1132 PMC4656347

[45] FDA Drug Safety Communication: Ongoing safety review of Recombinant Human Growth Hormone (somatropin) and possible increased risk of death. 2011. Available: http://www.fda.gov/Drugs/DrugSafety/ucm265865.htm

[46] Kawaguchi M, Kanemaru A, Fukushima T, Yamamoto K, Tanaka H, Haruyama Y, et al Ghrelin administration suppresses inflammation-associated colorectal carcinogenesis in mice. Cancer Sci. 2015 10.1111/cas.12725 PMC4582981 26094822

[47] Chen BJ, Deoliveira D, Spasojevic I, Sempowski GD, Jiang C, Owzar K, et al Growth hormone mitigates against lethal irradiation and enhances hematologic and immune recovery in mice and nonhuman primates. PLoS One. 2010; 5(6):e11056 10.1371/journal.pone.0011056 20585403 PMC2886847

[48] Wang Z, Lang Yang W, Jacob A, Aziz M, Wang P. Human ghrelin mitigates intestinal injury and mortality after whole body irradiation in rats. PLoS One. 2015; 10(2): e0118213 doi: 10.1371/journal. pone.0118213 25671547 10.1371/journal.pone.0118213 PMC4325005

[49] Sklar CA, Mertens AC, Mitby P, Occhiogrosso G, Qin J, Heller G, et al Risk of disease recurrence and second neoplasms in survivors of childhood cancer treated with growth hormone: a report from the Childhood Cancer Survivor Study. J Clin Endocrinol Metab. 2002;87: 3136–3141. 12107213 10.1210/jcem.87.7.8606

[50] Wilton P, Mattsson AF, Darendeliler F. Growth hormone treatment in children is not associated with an increase in the incidence of cancer: experience from KIGS (Pfizer International Growth Database). J Pediatr. 2010;157: 265–270. 10.1016/j.jpeds.2010.02.028 20400105

[51] Child CJ, Zimmermann AG, Woodmansee WW, Green DM, Li JJ, Jung H, et al Assessment of primary cancers in GH-treated adult hypopituitary patients: an analysis from the hypopituitary control and complications study. Eur J Endocrinol. 2011;165: 217–23. 10.1530/EJE-11-0286 21646285 PMC3132593

[52] Child CJ, Conroy D, Zimmermann AG, Woodmansee WW, Erfurth EM, Robinson LL. Incidence of primary cancers and intracranial tumour recurrences in GH-treated and untreated adult hypopituitary patients: analyses from the Hypopituitary Control and Complications Study. Eur J Endocrinol. 2015;172: 779–790. 10.1530/EJE-14-1123 25810462

[53] Shen L, Sun CM, Li XT, Liu CJ, Zhou YX. Growth hormone therapy and risk of recurrence/progression in intracranial tumors: a meta-analysis. Neurol Sci. 2015 10.1007/s10072-015-2269-z 26048536

[54] Carel JC, Ecosse E, Landier F, Meguellati-Hakkas D, Kaquelidou F, Rey G, et al Long-term mortality after recombinant growth hormone treatment for isolated growth hormone deficiency or childhood short stature: preliminary report of the French SAGhE study. J Clin Endocrinol Metab. 2012;97: 416–425. 10.1210/jc.2011-1995 22238382

[55] Perry JK, Emerald BS, Mertani HC, Lobie PE. The oncogenic potential of growth hormone. Growth Horm IGF Res. 2006;6: 277–289. 10.1016/j.ghir.2006.09.006 17101287

[56] Devesa J, Devesa P, Reimunde P. Growth hormone revisited. Med Clin (Barc). 2010;135: 665–670. 20045134 10.1016/j.medcli.2009.10.017

[57] Zhu T, Starling-Emerald B, Zhang X, Lee K, Gluckman PD, Mertani HC, et al Oncogenic transformation of human mammary epithelial cells by autocrine human growth hormone. Cancer Res. 2005;65: 317–324. 15665309

[58] Liang D, Zhang Y, Chen J, Wang H, Huang T, Xue X. Effects of exogenous growth hormone on Growth Hormone-Insulin-Like growth factor axis of human gastric cancer cell. Chin Med. 2014;5: 259–269.

[59] Xu XQ, Emerakd BS, Goh EL, Kanan N, Miller LD, Gluckman PD, et al Gene expression profiling to identify oncogenic determinants of autocrine human growth hormone in human mammary carcinoma. J Biol Chem. 2005;280: 23987–24003. 15845533 10.1074/jbc.M503869200

[60] Ibrahim YH, Yee D. Insulin-like growth factor-I and cancer risk. Growth Horm IGF Res. 2004;14: 261–269. 15231294 10.1016/j.ghir.2004.01.005

[61] Glynn N, Agha A. Diagnosing growth hormone deficiency in adults. Int J Endocrinol. 2012; 10.1155/2012/972617 PMC3412109 22899919

[62] Gomez de Segura IA, Vallejo-Cremades MT, Lomas J, Sanchez M, Caballero I, Largo C, et al Exogenous ghrelin regulates proliferation and apoptosis in the hypotrophic gut mucosa of the rat. Exp Biol Med. 2010;235: 463–469. 10.1258/ebm.2009.009247 20407078

